# Supplementary material for: A Synthetic Epoxydocosapentaenoic Acid Analogue Ameliorates Cardiac Ischemia/Reperfusion Injury: The Involvement of the Sirtuin 3–NLRP3 Pathway
Source: Int J Mol Sci. 2020 Jul 24;21(15):5261. doi: 10.3390/ijms21155261 (PMC7432620; doi:10.3390/ijms21155261)
Supplement: Supplementary file 1 [file ijms-21-05261-s001.pdf]

Supplementary Material for

A Synthetic Epoxydocosapentaenoic Acid Analogue Ameliorates Cardiac Ischemia/Reperfusion Injury: The Involvement of The Sirtuin 3 - NLRP3 Pathway

Ahmed M. Darwesh, Wesam Bassiouni, Adeniyi Michael Adebesin, Abdul Sattar Mohammad, John R. Falck and John M. Seubert

Method S1. Synthesis of SA-26.

Synthesis of methyl docos-16(Z)-en-4-ynoate.

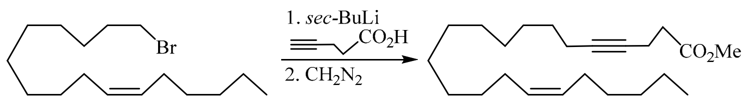

To a stirring, -78 °C solution of 4-pentynoic acid (GSF, 341 mg, 3.47 mmol) in anhydrous THF/HMPA (4:1, 10 mL) under an argon atmosphere was added dropwise *sec*-BuLi (5.79 mL, 1.4 M hexane solution). The reaction mixture was warmed over 40 min to 0 °C. After an additional 3 h at the same temperature, a solution of 16-bromohexadec-6(Z)-ene[1] (920 mg, 2.89 mmol) in THF (5 mL) was added and the whole was allowed to stir at rt overnight. Following quenching with aq. 1 N HCl, the reaction mixture was extracted with EtOAc (3 × 50 mL) and the combined extracts were dried over Na_2SO_4, filtered, and concentrated *in vacuo*. The crude was treated with excess diazomethane in Et_2O at 0 ° for 2 h, then purified by SiO_2 column chromatography to give methyl docos-16(Z)-en-4-ynoate (465 mg, 48% over 2 steps) as a colorless oil. TLC: R_f ~ 0.43 (5% EtOAc/hexanes). ^{1}H NMR (300 MHz, $CDCl_3$) δ 5.43–5.26 (m, 2H), 3.69 (s. 3H), 2.57–2.41 (m, 4H), 2.17–2.06 (m, 2H), 2.06–1.92 (m, 4H), 1.53–1.18 (m, 22H), 0.88 (t, *J* = 6.6 Hz, 3H).

Synthesis of methyl 15-(3-pentyloxiran-2-yl)pentadec-4-ynoate.

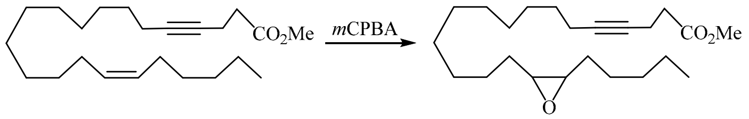

m-Chloroperbenzoic acid (77%, 132 mg, 0.76 mmol) was added portionwise to a stirring, 0 °C solution of the above ester (223 mg, 0.64 mmol) in anhydrous CH_2Cl_2 (5 mL). After 1 h at rt, the reaction mixture was quenched with sat. Na_2SO_3 solution, extracted with EtOAc, and concentrated and the residue purified by SiO_2 column chromatography to give methyl 15-(3-pentyloxiran-2-yl)pentadec-4-ynoate (191 mg, 82%) as an oil. TLC: R_f ~ 0.5 (10% EtOAc/hexanes). ^{1}H NMR (300 MHz, $CDCl_3$) δ 3.69 (s, 3H), 2.90 (t, *J* = 3 Hz, 2H), 2.57–2.38 (m, 4H), 2.16–2.04 (m, 2H), 1.58–1.16 (m, 28H), 0.89 (t, *J* = 6.6 Hz, 3H).

Synthesis of methyl 15-(3-pentyloxiran-2-yl)pentadec-4(Z)-enoate.

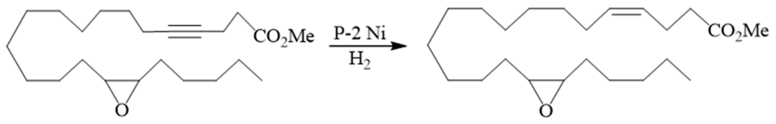

NaBH$_4$ (10 mg, 0.27 mmol) was added to stirring solution of Ni(OAc)$_2$·4H$_2$O (68 mg, 0.27 mmol) in absolute EtOH (5 mL) under a hydrogen atmosphere (1 atm). After 10 min, distilled ethylenediamine (25 mg, 0.54 mmol) was added to the resultant black suspension followed after another 10 min by a solution of methyl 15-(3-pentyloxiran-2-yl)pentadec-4-ynoate in ab. EtOH (2 mL). After 1 h, the reaction mixture was passed through a bed of SiO$_2$ that was washed by EtOAc (5 mL). The combined eluates were concentrated and purified via SiO$_2$ column chromatography to give methyl 15-(3-pentyloxiran-2-yl)pentadec-4(Z)-enoate (188 mg, 98%) as an oil. TLC: R_f ~ 0.52 (10% EtOAc/hexanes). ^{1}H NMR (300 MHz, CDCl$_3$) δ 5.43–5.29 (m, 2H), 3.66 (s, 3H), 2.89 (t, *J* = 3 Hz, 2H), 2.35–2.34 (m, 4H), 2.03–1.99 (m, 2H), 1.48–1.26 (m, 24H), 0.89 (t, *J* = 6.6 Hz, 3H); ^{13}C NMR (CDCl$_3$, 75 MHz) δ 173.75, 131.69, 127.37, 57.31, 51.60, 34.27, 31.85, 29.75, 29.71, 29.68, 29.66, 29.41, 27.95, 27.91, 27.30, 26.73, 26.41, 22.91, 22.72, 13.11.

Synthesis of (*Z*)-15-(3-pentyloxiran-2-yl)pentadec-4-enoic acid (SA-26).

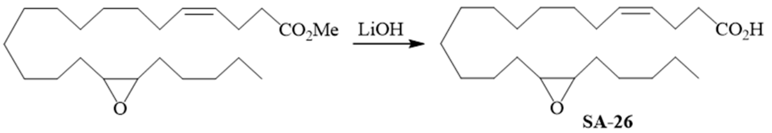

SA-26

To a solution of methyl 15-(3-pentyloxiran-2-yl)pentadec-4(Z)-enoate (119 mg, 0.32 mmol) in THF/H$_2$O (4:1, 5 mL) was added LiOH (1 m aq. soln, 1 mL) at 0 °C. After stirring at rt for 12 h, the mixture was acidified to pH 4.5 using 1 M aq. oxalic acid and extracted with EtOAc (3 × 20 mL). The combined extracts were washed with brine, concentrated *in vacuo*, and the residue purified via SiO$_2$ chromatography to give **SA-26** (100 mg, 88%) as a colorless oil. TLC: R_f ~ 0.45 (30% EtOAc/hexanes). ^{1}H NMR (300 MHz, CDCl$_3$) δ 5.47–5.42 (m, 1H), 5.37–5.32 (m, 1H), 2.92 (t, *J* = 3.3, 2H), 2.41–2.35 (m, 4H), 2.06–2.01 (m, 2H), 1.50–1.27 (m, 26H), 0.90 (t, *J* = 5.4 Hz, 3H).

Method S2. Synthesis of AS-27

Synthesis of 18-((*tert*-butyldiphenylsilyl)oxy)octadec-5(Z)-en-1-amine.

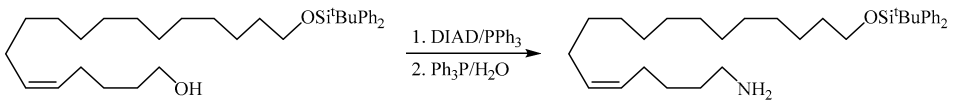

Diphenylphosphoryl azide (DIAD, 1.15 g, 4.1 mmol) was added dropwise to a 0 °C solution of the known[2] 18-((*tert*-butyldiphenylsilyl)oxy)octadec-5(Z)-en-1-ol (1.82 g, 3.4 mmol) and Ph$_3$P (1.02 g, 4.1 mmol) in anhydrous THF (50 mL). After stirring at rt for 22 h, water (10 mL) was added and the reaction mixture was extracted with EtOAc (2 × 20 mL). The combined extracts were dried over Na$_2$SO$_4$, filtered, and concentrated *in vacuo*. The residue was used for next step without further purification.

Ph$_3$P (0.9 g, 3.54 mmol) was added to a stirring, rt solution of the above crude azide (1.82 g, 2.3 mmol) in anhydrous THF (30 mL). After 2 h, water (2 mL) was added and allowed to stir an additional 48 h. The reaction mixture was diluted with water (10 mL), extracted with EtOAc (2 × 30 mL), and the combined extracts were dried over Na$_2$SO$_4$, filtered, and concentrated *in vacuo*. Purification of the residue by SiO$_2$ column chromatography using a gradient of 25-35% EtOAc/hexanes afforded 18-

((*tert*-butyldiphenylsilyl)oxy)octadec-5(*Z*)-en-1-amine (1.06 g, 71% over 2 steps) as an oil. TLC: R_f ~ 0.2 (50% EtOAc/hexanes). ^{1}H NMR (400 MHz, $CDCl_3$) δ 7.67 (dt, *J* = 7.8, 1.6 Hz, 4H), 7.40 (dt, *J* = 10.7, 1.5 Hz, 6H), 5.44–5.31 (m, 2H), 3.65 (td, *J* = 6.5, 1.4 Hz, 2H), 2.72 (t, *J* = 7.0 Hz, 2H), 2.24–1.94 (m, 6H), 1.63–1.18 (m, 24H), 1.05 (d, *J* = 1.4 Hz, 9H); ^{13}C NMR (101 MHz, $CDCl_3$) δ 135.56, 134.16, 130.35, 129.45, 129.30, 127.54, 64.01, 32.59, 29.76, 29.70, 29.67, 29.63, 29.59, 29.40, 29.35, 27.26, 26.99, 26.96, 26.86, 26.78, 25.77, 19.22.

Synthesis of 1-(18-((*tert*-butyldiphenylsilyl)oxy)octadec-5(*Z*)-en-1-yl)-3-ethylurea.

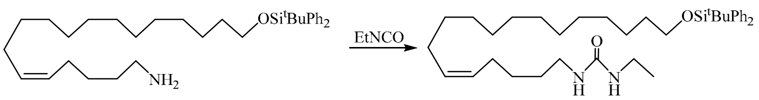

A solution of the above amine (0.75 g, 1.36 mmol) and EtNCO (0.1 g, 1.4 mmol) was stirred at rt in dry THF (20 mL) for 18 h, then all volatiles were removed *in vacuo*. The residue was purified by SiO_2 column chromatography using 5% methanol/CH_2Cl_2 and dichloromethane as eluent to provide 1-(18-((*tert*-butyldiphenylsilyl)oxy)octadec-5(*Z*)-en-1-yl)-3-ethylurea (0.6 g, 79 % yield) as an oil. TLC: R_f ~ 0.3 (50% EtOAc/hexanes). ^{1}H NMR (400 MHz, CDCl3) δ 7.74–7.55 (m, 4H), 7.47–7.29 (m, 6H), 5.42–5.23 (m, 2H), 4.18 (s, 2H), 3.65 (t, *J* = 6.5 Hz, 2H), 3.21–3.19 (m, 4H), 2.05–2.02 (m, 4H), 1.66–1.17 (m, 24H), 1.13 (t, *J* = 7.2 Hz, 3H), 1.04 (s, 9H); ^{13}C NMR (101 MHz, $CDCl_3$) δ 170.56, 135.24, 133.85, 130.19, 129.13, 128.77, 127.21, 63.69, 40.24, 35.10, 32.26, 29.50, 29.43, 29.35, 29.30, 29.26, 29.07, 29.03, 26.95, 26.64, 26.54, 26.50, 25.45, 18.89, 15.16.

Synthesis of 1-ethyl-3-(18-hydroxyoctadec-5(*Z*)-en-1-yl)urea.

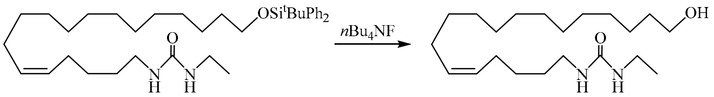

TBAF (1 M THF soln, 1 mL, 1.04 mmol) was added to a stirring, 0 °C solution of the above urea (0.65 g, 1.04 mmol) in dry THF (15 mL). After stirring at rt for 12 h, water (10 mL) was added and the reaction mixture was extracted with EtOAc (2 × 20 mL). The combined extracts were dried over Na_2SO_4, filtered, and concentrated *in vacuo*. The residue was purified SiO_2 column chromatography using a gradient of 30-45% EtOAc/hexanes to give 1-ethyl-3-(18-hydroxyoctadec-5(*Z*)-en-1-yl)urea (300 mg, 81%) as a white semi-solid. TLC: R_f ~0.5 (80% EtOAc/hexanes). ^{1}H NMR (400 MHz, $CDCl_3$) δ 5.41–5.28 (m, 2H), 4.30 (d, *J* = 8.2 Hz, 2H), 3.63 (t, *J* = 6.7 Hz, 2H), 3.23–3.13 (m, 4H), 2.01 (dt, *J* = 5.6, 7.0 Hz, 4H), 1.60–1.44 (m, 5H), 1.43–1.22 (m, 21H), 1.13 (t, *J* = 7.2 Hz, 3H); ^{13}C NMR (101 MHz, $CDCl_3$) δ 158.14, 130.48, 129.12, 63.05, 40.53, 35.39, 32.79, 29.84, 29.66, 29.56, 29.52, 29.46, 29.40, 29.23, 27.20, 26.98, 26.83, 25.72, 15.47.

Synthesis of 18-(3-ethylureido)octadec-13(*Z*)-enoic acid (AS-27).

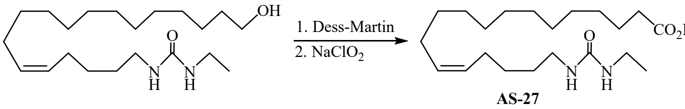

AS-27

Dess-Martin periodinane (167 mg, 0.39 mmol) and $NaHCO_3$ (44 mg, 0.52 mmol) were added sequentially to a stirring, 0 °C solution of the above alcohol (100 mg, 0.26 mmol) in CH_2Cl_2 (20 mL). After stirring for 3 h at rt, the reaction mixture was quenched with a saturated aq. solution of sodium thiosulfate (10 mL) and extracted with CH_2Cl_2 (2 × 20 mL). The combined extracts were dried over Na_2SO_4, concentrated *in vacuo* and the crude 1-ethyl-3-(18-oxooctadec-5(*Z*)-en-1-yl)urea was used in the next step without purification.

To a stirring, 0 °C solution of the above aldehyde (80 mg, 0.21 mmol) in *tert*-BuOH and water (1:1, 8 mL) were added sequentially 2-methyl-2-butene (147 mg, 2.1 mmol), NaH_2PO_4 (58 mg, 0.42 mmol), and $NaClO_2$ (28 mg, 0.31 mmol). After 4 h, saturated aq. NH_4Cl (10 mL) added to the reaction mixture which was then extracted with EtOAc (2 × 20 mL). The combined organic extracts were dried over Na_2SO_4, concentrated *in vacuo* and the residue purified by SiO_2 column chromatography using

a gradient of 5-10% methanol/CH_2Cl_2 to give 18-(3-ethylureido)octadec-13(*Z*)-enoic acid (**AS-27**, 54 mg, 63% over two steps) as a white powder. TLC: R_f ~ 0.5 (10% MeOH/CH_2Cl_2. ^{1}H NMR (400 MHz, $CDCl_3$) δ 5.45–5.26 (m, 2H), 4.93–4.40 (m, 2H), 3.20 (q, *J* = 7.3 Hz, 2H), 3.10 (t, *J* = 7.1 Hz, 2H), 2.32 (t, *J* = 7.1 Hz, 2H), 2.05–2.01 (m, 4H), 1.57 (dq, *J* = 6.3, 7.2 Hz, 4H), 1.44–1.21 (m, 18H), 1.15 (t, *J* = 7.2 Hz, 3H); ^{13}C NMR (101 MHz, $CDCl_3$) δ 177.75, 159.14, 130.58, 129.09, 77.32, 77.00, 76.68, 40.80, 35.46, 34.06, 29.36, 29.34, 29.09, 28.93, 28.87, 28.84, 28.78, 28.71, 26.87, 26.79, 24.68, 15.27.

Method S3. Synthesis of (*Z*)-15-(3-pentyloxiran-2-yl)pentadec-7-enoic acid (AA-4).

This analog was prepared from the known[1] 1-bromoundec-5(*Z*)-ene as described for **SA-26** in 10% overall yield.

^{1}H NMR (500 MHz, C_6D_6) δ 5.57 – 5.33 (m, 2H), 2.84 – 2.73 (m, 2H), 2.18 – 1.90 (m, 7H), 1.60 – 1.08 (m, 31H), 0.87 (t, *J* = 7.0 Hz, 3H).

Spectra

Figure S1. Spectrum of methyl docos-16(*Z*)-en-4-ynoate.

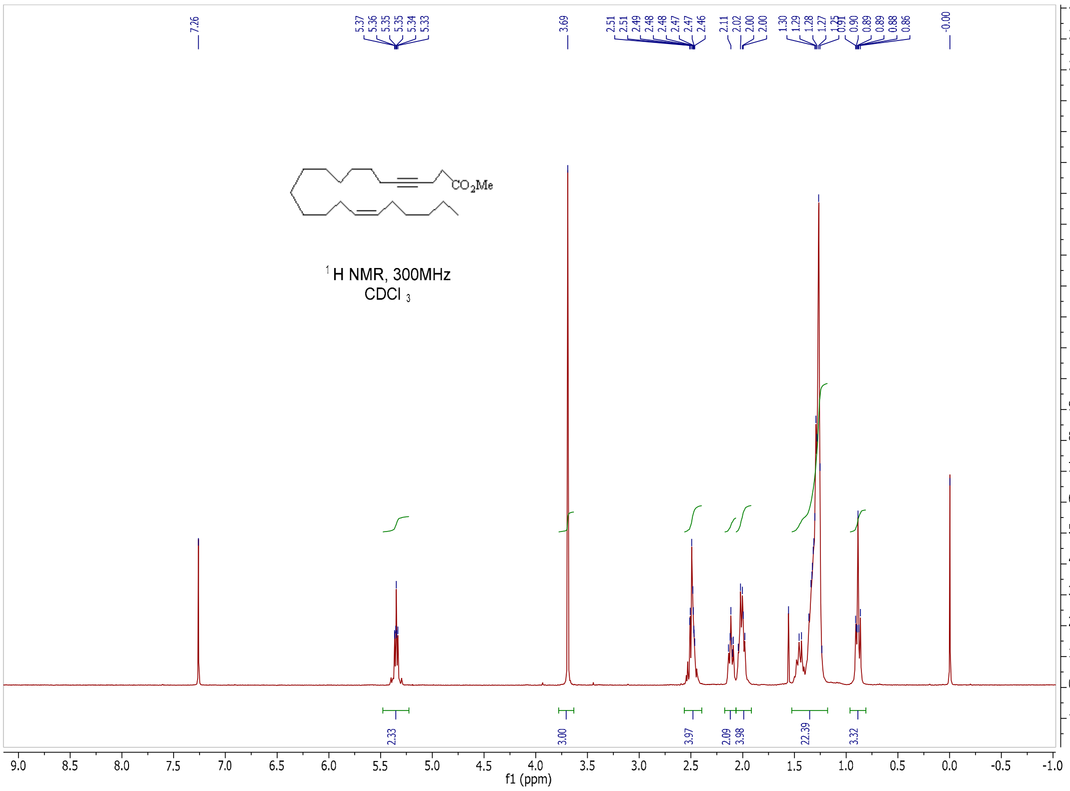

Figure S2. Spectrum of methyl 15-(3-pentyloxiran-2-yl)pentadec-4-ynoate.

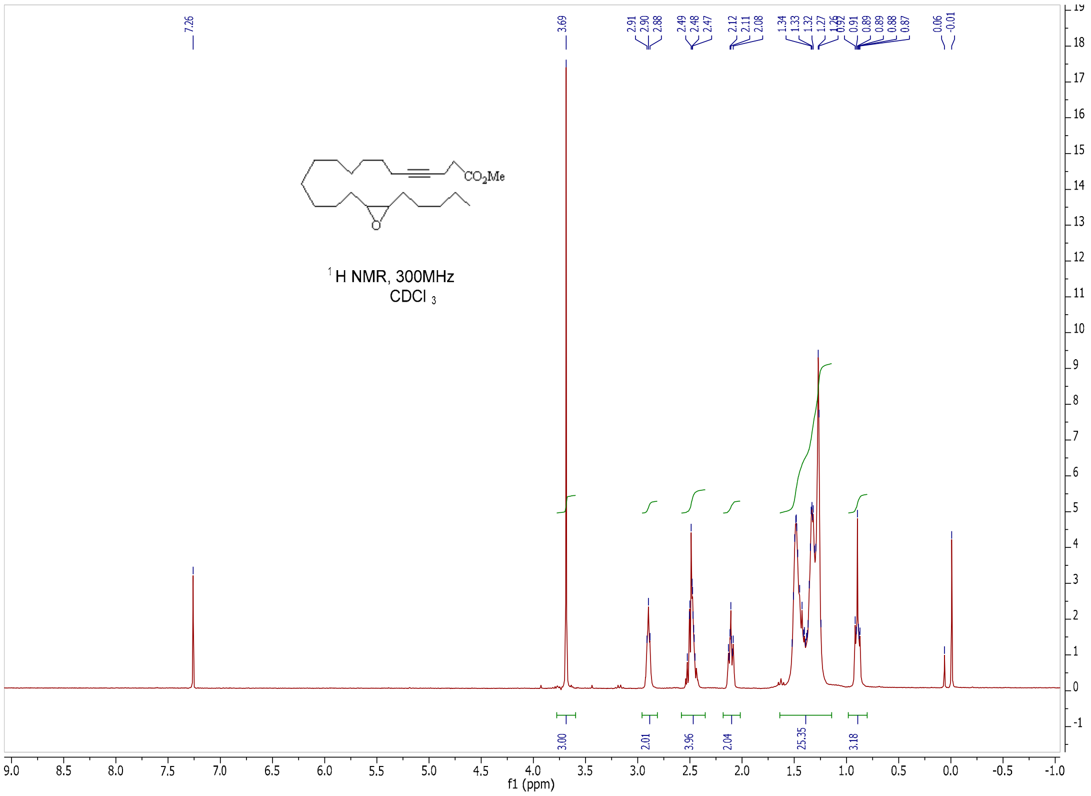

Figure S3. Spectra of methyl 15-(3-pentyloxiran-2-yl)pentadec-4(*Z*)-enoate.

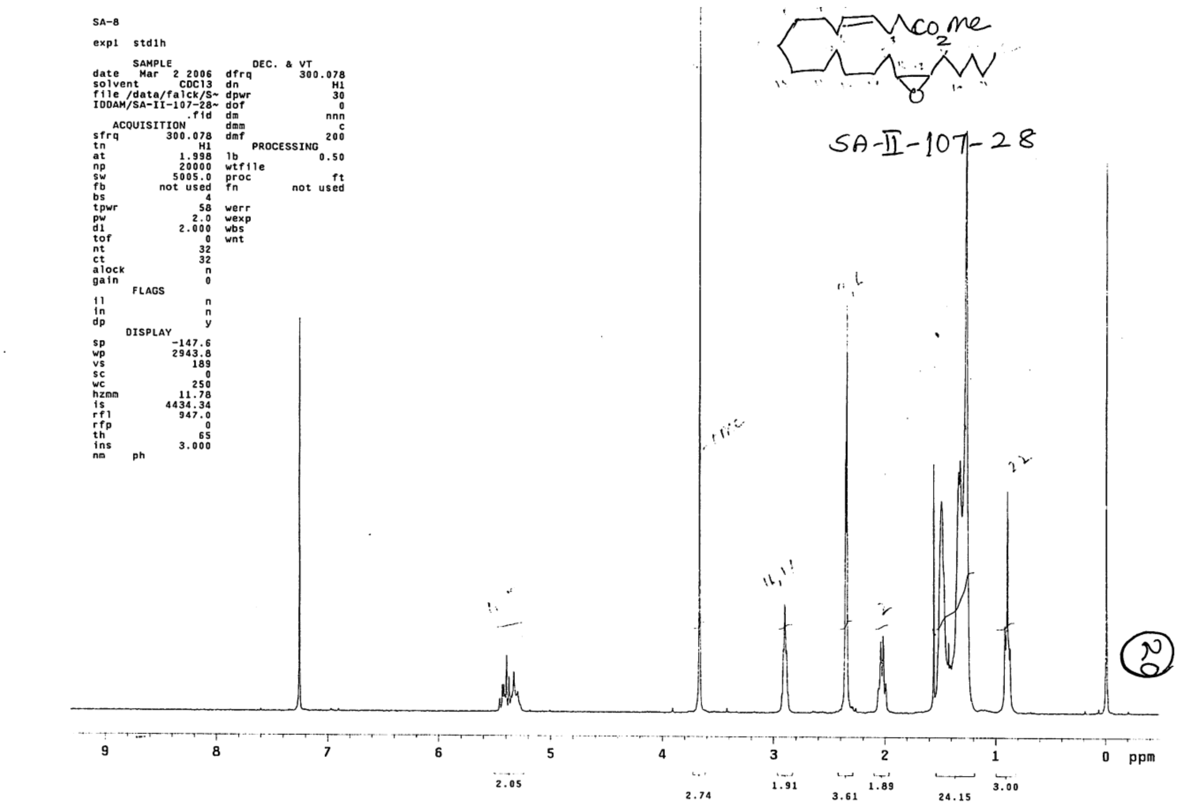

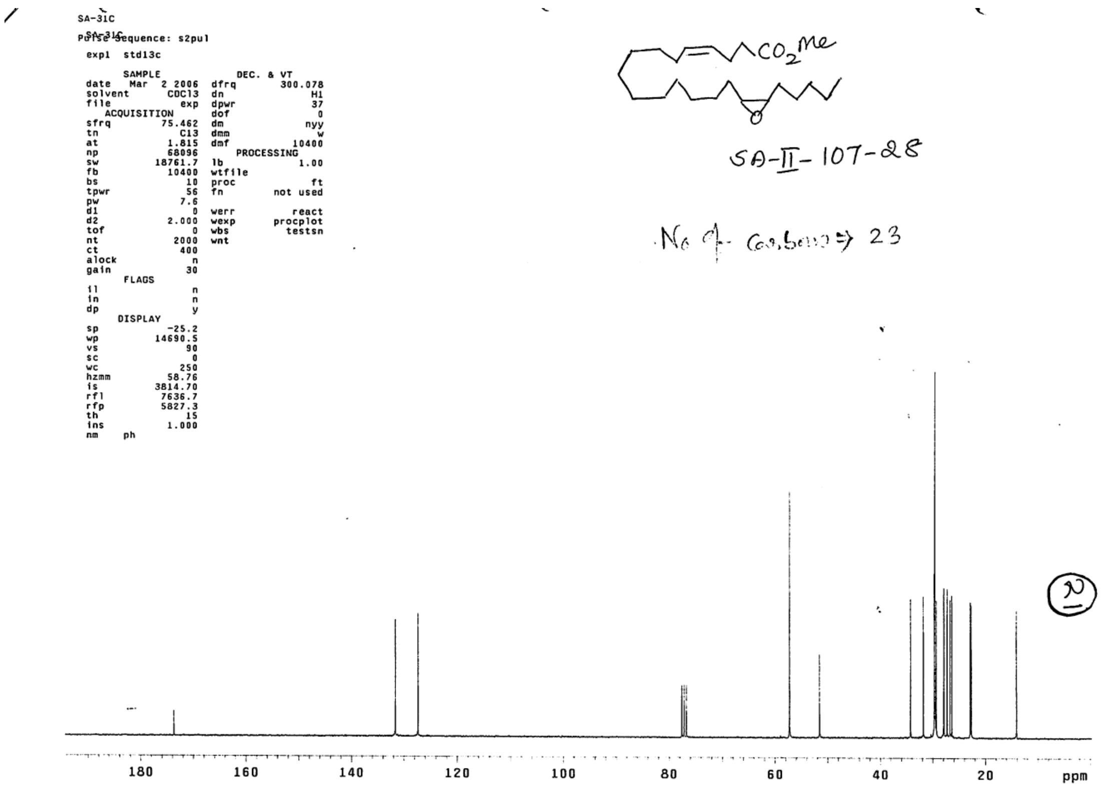

Figure S4. Spectrum of (*Z*)-15-(3-pentyloxiran-2-yl)pentadec-4-enoic acid (SA-26).

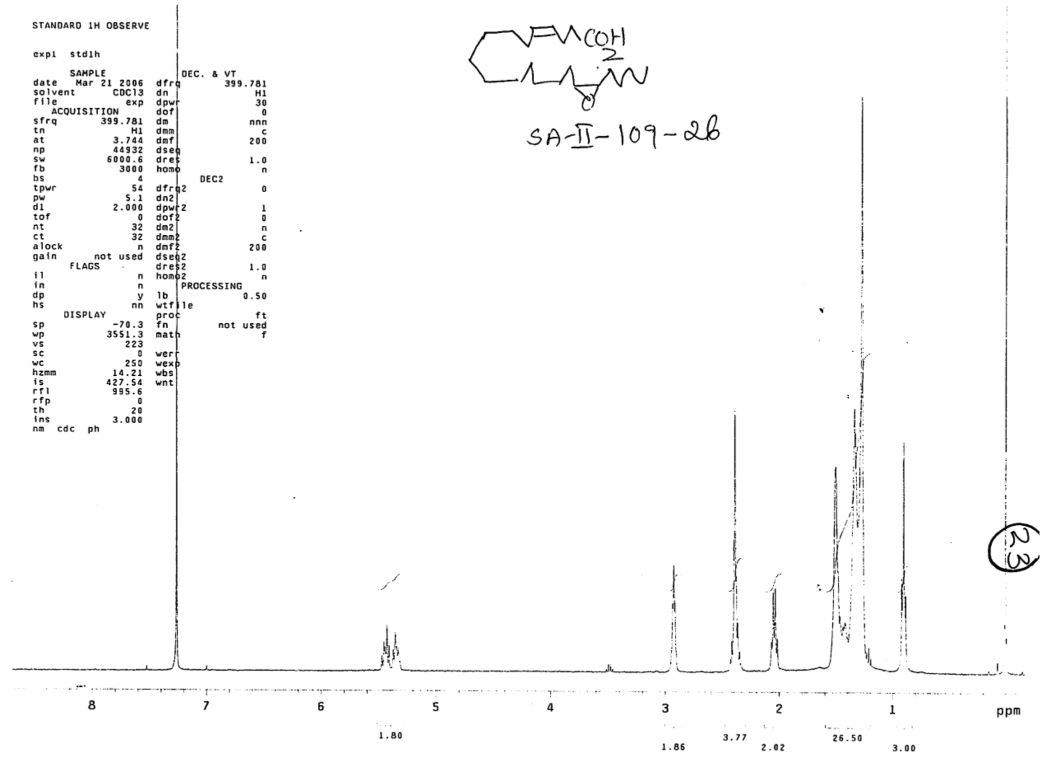

Figure S5. Spectra of methyl docos-16(*Z*)-en-4-ynoate.

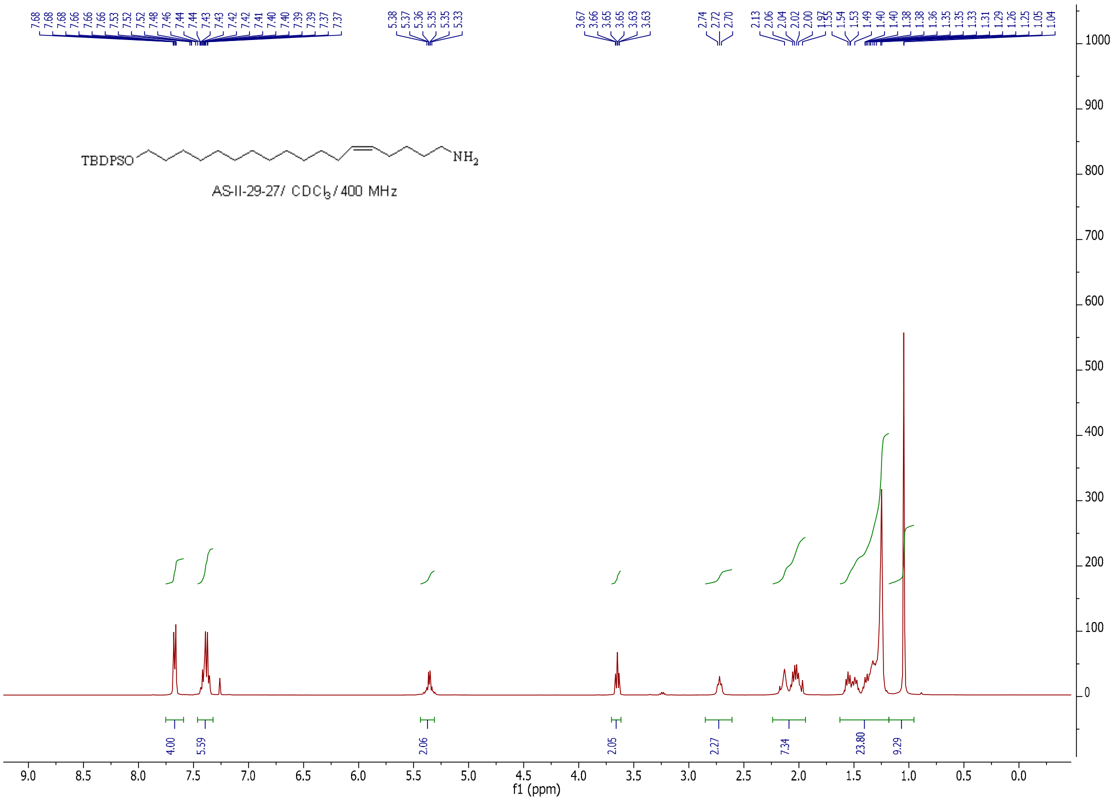

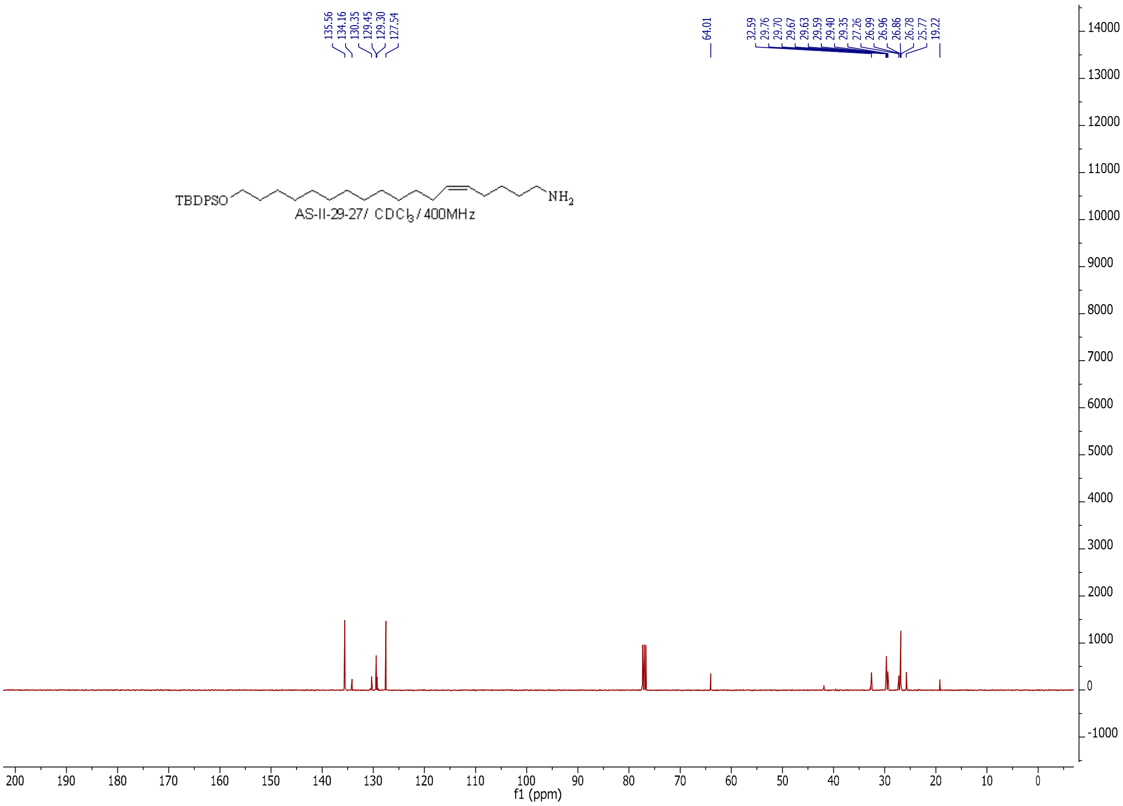

Figure S6. Spectra of 1-(18-((*tert*-butyldiphenylsilyl)oxy)octadec-5(*Z*)-en-1-yl)-3-ethylurea.

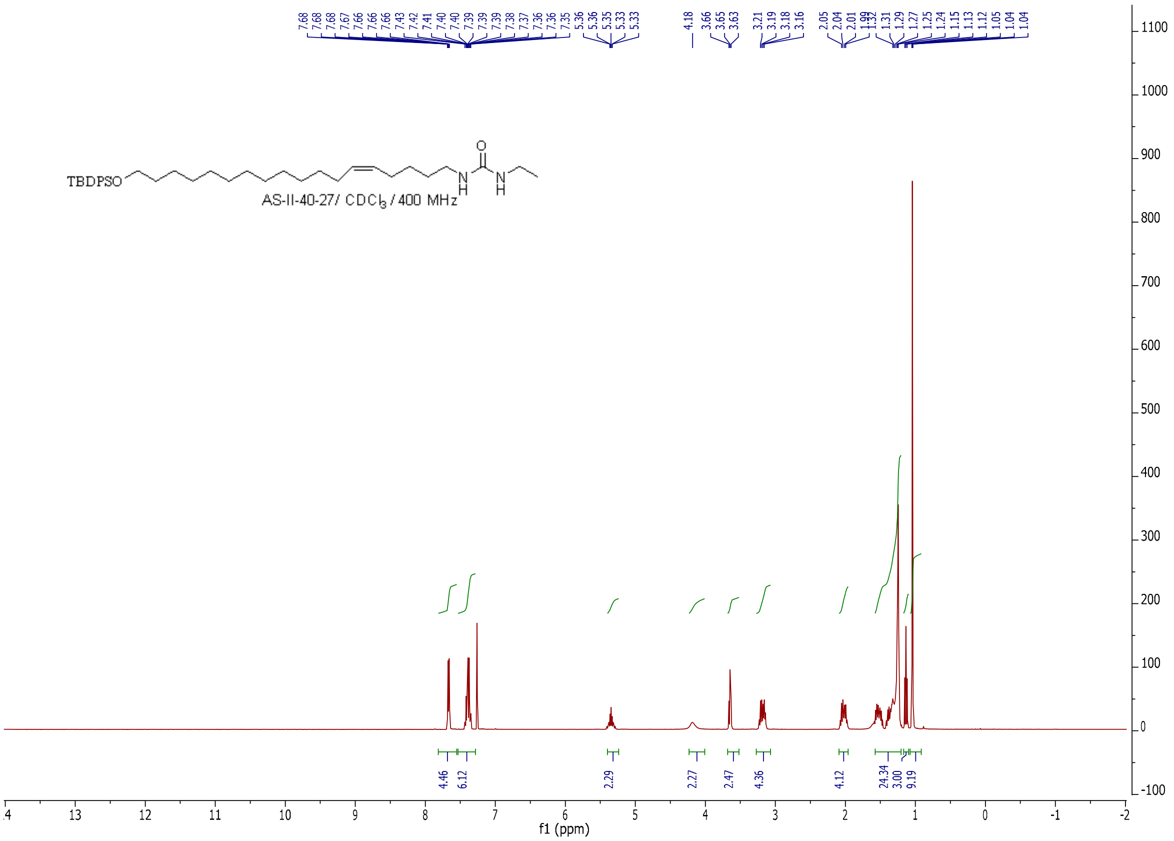

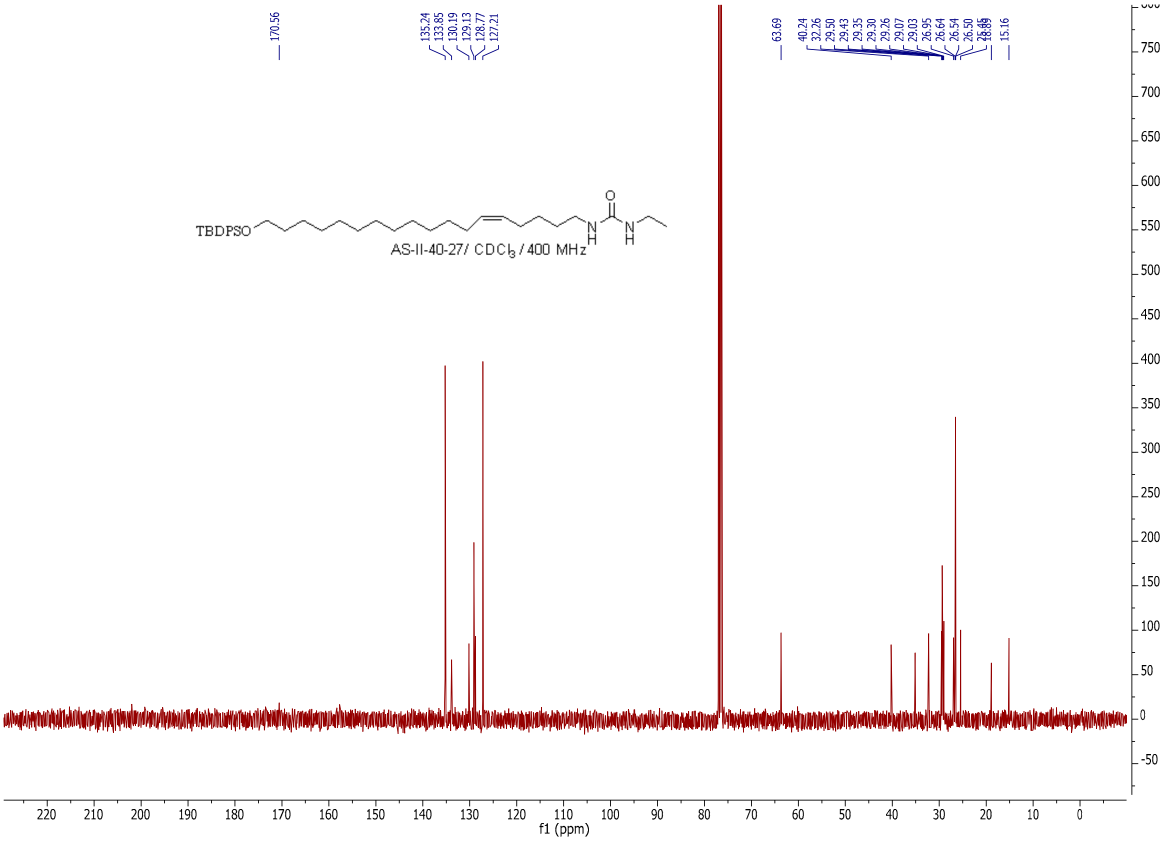

Figure S7. Spectra of 1-ethyl-3-(18-hydroxyoctadec-5(*Z*)-en-1-yl)urea.

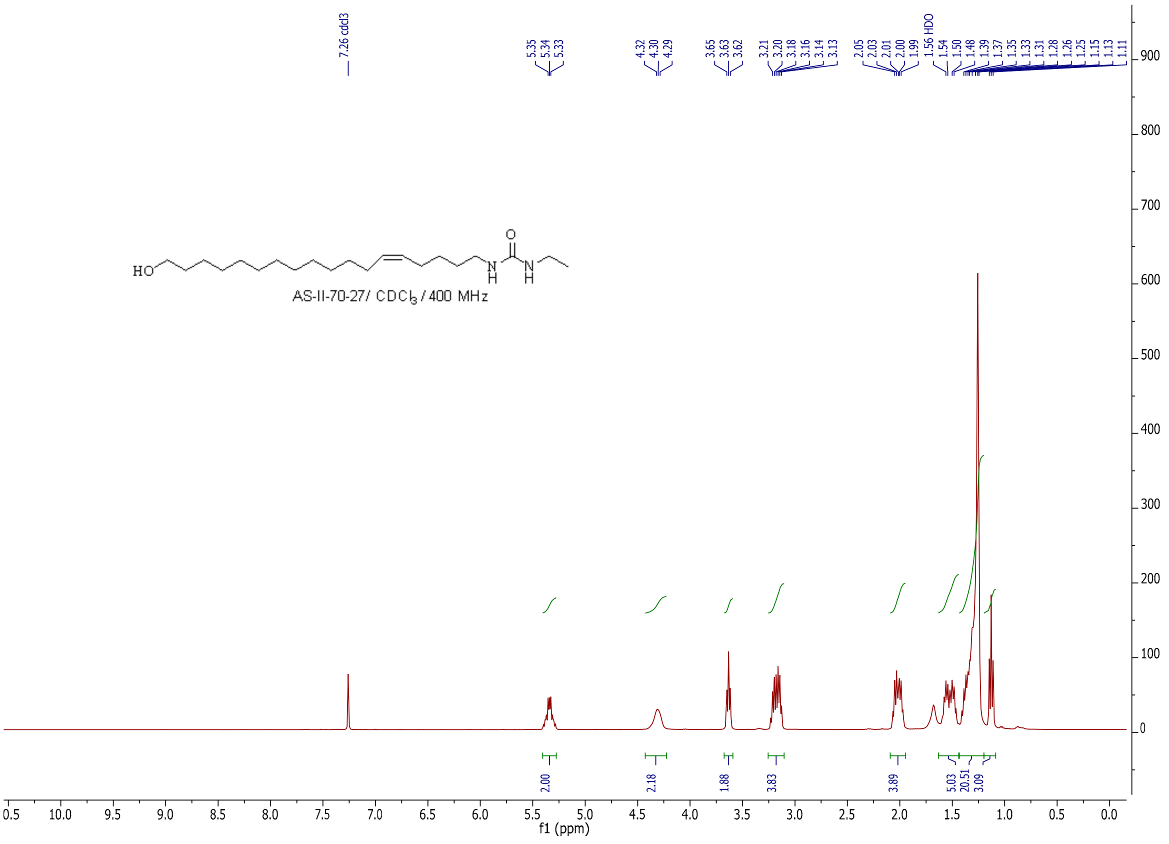

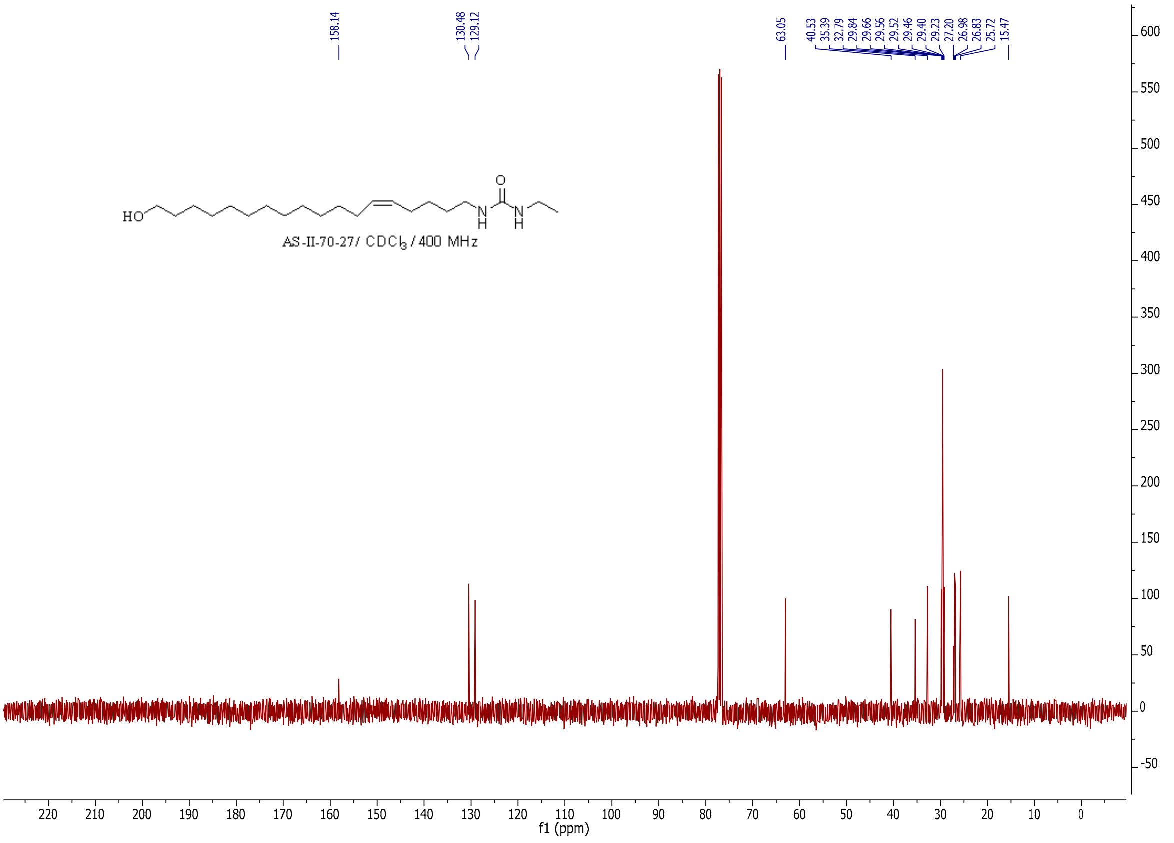

Figure S8. Spectra of 18-(3-ethylureido)octadec-13(*Z*)-enoic acid (AS-27).

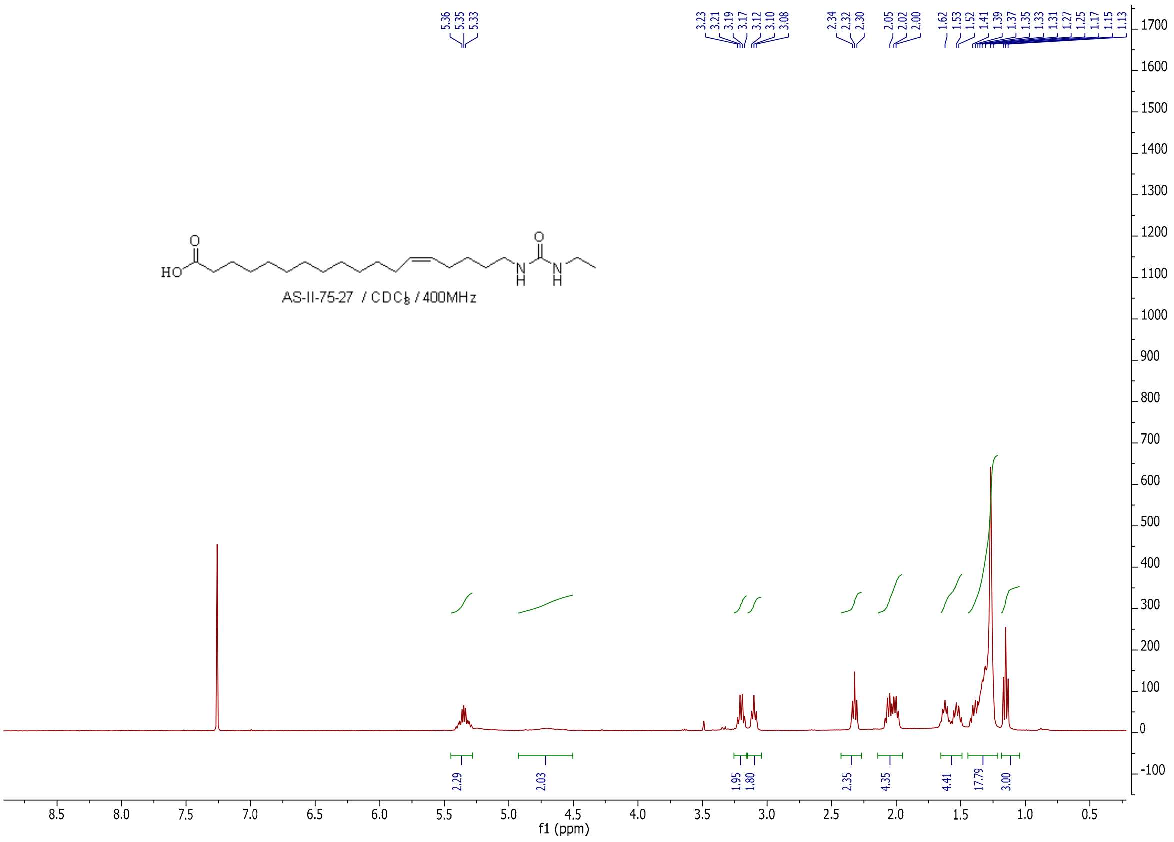

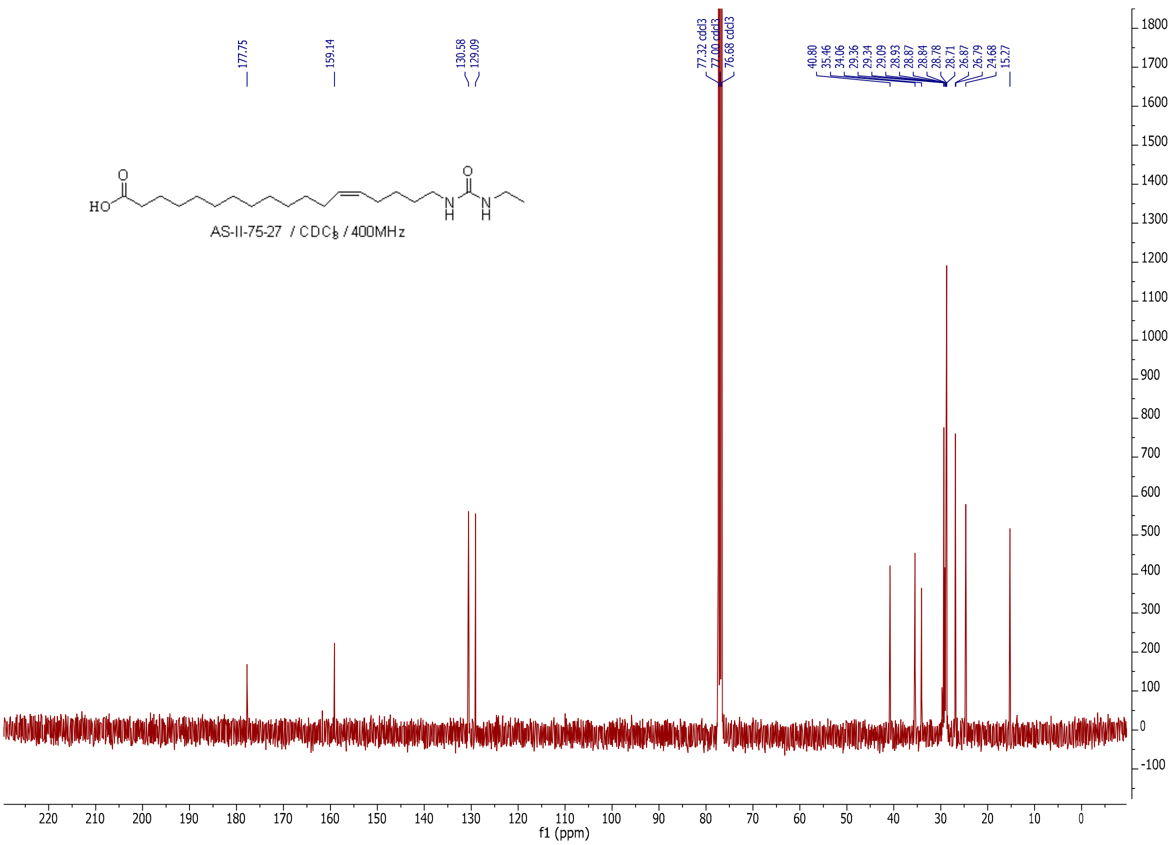

Figure S9: Spectrum of (*Z*)-15-(3-pentyloxiran-2-yl)pentadec-7-enoic acid (AA-4).

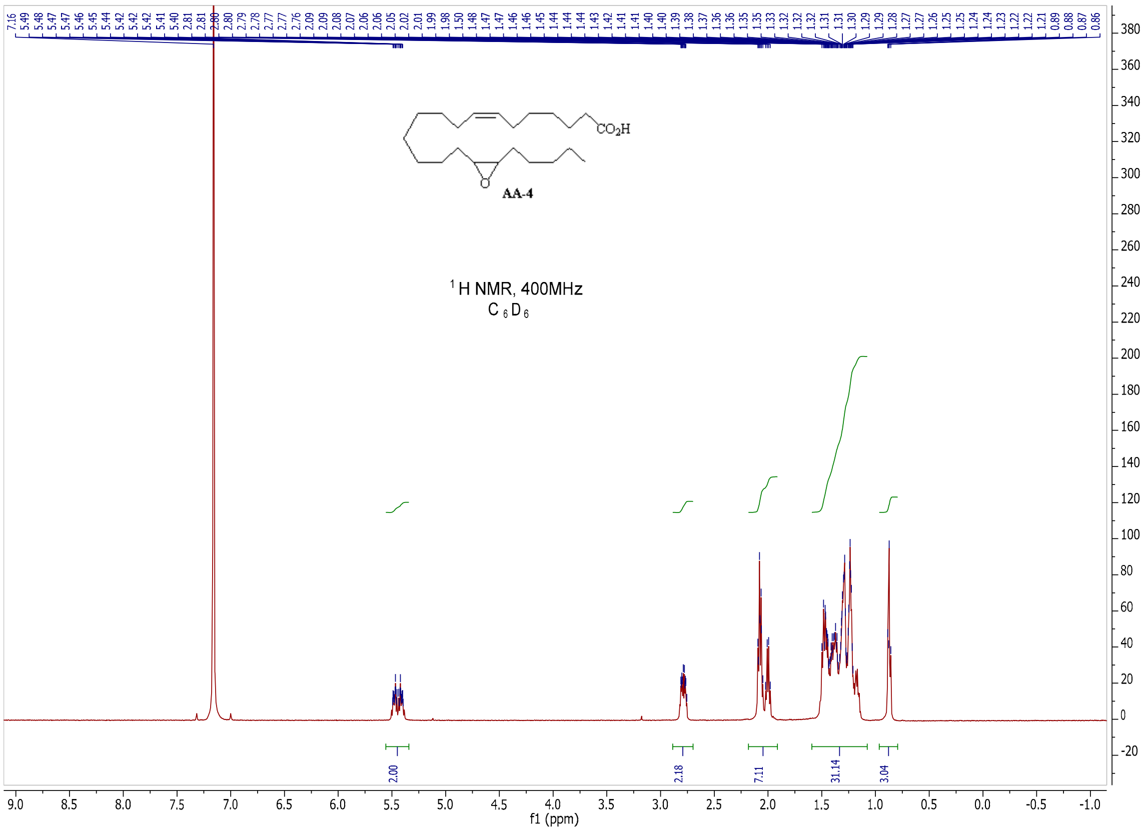

References

1. Zhang, H.-Y.; Yamakawa, Y.-i.; Matsuya, Y.; Toyooka, N.; Tohda, C.; Awale, S.; Li, F.; Kadota, S.; Tezuka, Y., Synthesis of Long-Chain Fatty Acid Derivatives as a Novel Anti-Alzheimer's Agent. *Bioorg. Med. Chem. Lett*. **24**: 604-608, 2014.
2. Falck, John R.; Koduru, Sreenivasulu Reddy; Mohapatra, Seetaram; Manne, Rajkumar; Atcha, Krishnam Raju; Manthati, Vijaya L.; Capdevila, Jorge H.; Christian, Sarah; Imig, John D.; Campbell, William B., 14,15-Epoxyeicosa-5,8,11-trienoic Acid (14,15-EET) Surrogates: Carboxylate Modifications. *J. Med. Chem.* **57**: 6965-6972, 2014.
